# New COVID-19 Cases and Hospitalizations Among Adults, by Vaccination Status — New York, May 3–July 25, 2021

**DOI:** 10.15585/mmwr.mm7034e1

**Published:** 2021-08-27

**Authors:** Eli S. Rosenberg, David R. Holtgrave, Vajeera Dorabawila, MaryBeth Conroy, Danielle Greene, Emily Lutterloh, Bryon Backenson, Dina Hoefer, Johanne Morne, Ursula Bauer, Howard A. Zucker

**Affiliations:** ^1^New York State Department of Health; ^2^University at Albany School of Public Health, State University of New York, Rensselaer, New York.

Data from randomized clinical trials and real-world observational studies show that all three COVID-19 vaccines currently authorized for emergency use by the Food and Drug Administration* are safe and highly effective for preventing COVID-19–related serious illness, hospitalization, and death (*1*,*2*). Studies of vaccine effectiveness (VE) for preventing new infections and hospitalizations attributable to SARS-CoV-2, the virus that causes COVID-19), particularly as the B.1.617.2 (Delta) variant has become predominant, are limited in the United States (*3*). In this study, the New York State Department of Health linked statewide immunization, laboratory testing, and hospitalization databases for New York to estimate rates of new laboratory-confirmed COVID-19 cases and hospitalizations by vaccination status among adults, as well as corresponding VE for full vaccination in the population, across all three authorized vaccine products. During May 3–July 25, 2021, the overall age-adjusted VE against new COVID-19 cases for all adults declined from 91.7% to 79.8%. During the same period, the overall age-adjusted VE against hospitalization was relatively stable, ranging from 91.9% to 95.3%. Currently authorized vaccines have high effectiveness against COVID-19 hospitalization, but effectiveness against new cases appears to have declined in recent months, coinciding with the Delta variant’s increase from <2% to >80% in the U.S. region that includes New York and relaxation of masking and physical distancing recommendations. To reduce new COVID-19 cases and hospitalizations, these findings support the implementation of a layered approach centered on vaccination, as well as other prevention strategies such as masking and physical distancing.

Four databases (the Citywide Immunization Registry, New York State Immunization Information System, Electronic Clinical Laboratory Reporting System, and Health Electronic Response Data System [HERDS]) were linked to construct a surveillance-based cohort of adults aged ≥18 years residing in New York by using individual name-based identifiers, date of birth, and zip code of residence. The Citywide Immunization Registry and the New York State Immunization Information System are used to collect and store all COVID-19 provider vaccination data for persons residing in New York City and the rest of the state, respectively (excluding selected settings such as Veterans Affairs and military health care facilities); persons were considered fully vaccinated ≥14 days after receipt of the final vaccine dose.^†^ The Electronic Clinical Laboratory Reporting System collects all reportable COVID-19 test results (nucleic acid amplification test [NAAT] or antigen) in New York (*4*); a new COVID-19 case was defined as the receipt of a new positive SARS-CoV-2 NAAT or antigen test result, but not within 90 days of a previous positive result. HERDS includes a statewide, daily electronic survey of all inpatient facilities in New York; new admissions with a laboratory-confirmed COVID-19 diagnosis are entered into HERDS daily by trained hospital staff members.

After a period of phased COVID-19 vaccine eligibility based on age, occupation, setting, or comorbidities beginning in December 2020, all New York residents aged ≥60 years were eligible for vaccination by March 10, 2021; eligibility was expanded to persons aged ≥30 years by March 30, and to all adults aged ≥18 years by April 6.^§^ To allow time for a large portion of vaccinated persons to achieve full immunity, this study was restricted to the week beginning May 3 through the week beginning July 19, 2021.

Breakthrough infections were defined as new cases among persons who were fully vaccinated on the day of specimen collection. Hospitalizations among persons with breakthrough infection were defined as new hospital admissions among persons fully vaccinated on the reporting day. The total adult state population that was fully vaccinated and unvaccinated^¶^ was assessed for each day and stratified by age group (18–49 years, 50–64 years, and ≥65 years). Persons who were partially vaccinated were excluded from analyses. For each week and age group, the rates of new cases and hospitalizations were calculated among fully vaccinated and unvaccinated persons, by respectively dividing the counts for each group by the fully vaccinated and unvaccinated person-days in that week. Age-adjusted VE each week was estimated as the population-weighted mean of the age-stratified VE.** The interval between completing vaccination and positive SARS-CoV-2 test result date was summarized using the median, interquartile range (IQR), and percentage tested ≥7 days from being fully vaccinated.^††^ The ratio of hospitalizations to cases was computed for each vaccination group to understand the relative severity of cases. Statistical testing was not performed because the study included the whole population of interest and was not a sample.

By July 25, 2021, a total of 10,175,425 (65.8%) New York adults aged ≥18 years were fully vaccinated; 1,603,939 (10.4%) were partially vaccinated. Among fully vaccinated adults, 51.3% had received Pfizer-BioNTech, 39.8% had received Moderna, and 8.9% had received Janssen (Johnson & Johnson) vaccines. During May 3–July 25, a total of 9,675 new cases (1.31 per 100,000 person-days) occurred among fully vaccinated adults, compared with 38,505 (10.69 per 100,000 person-days) among unvaccinated adults (Table). Most (98.1%) new cases among fully vaccinated persons occurred ≥7 days after being classified fully vaccinated (median = 85 days; IQR = 58–113). During May 3–July 25, case rates among fully vaccinated persons were generally similar across age groups, as were case rates among unvaccinated persons, declining through the end of June before increasing in July (Figure 1). Weekly estimated VE against new laboratory-confirmed infection during May 3–July 25 for all age groups generally declined, ranging from 90.6% to 74.6% for persons aged 18–49 years, 93.5% to 83.4% for persons aged 50–64 years, and 92.3% to 88.9% for persons aged ≥65 years. During May 3–July 25, the overall, age-adjusted VE against infection declined from 91.7% to 79.8% (Figure 1) (Table).

**TABLE:** Vaccination coverage, new COVID-19 cases, and new hospitalizations with laboratory-confirmed COVID-19 among fully vaccinated and unvaccinated adults, and estimated vaccine effectiveness — New York, May 3–July 25, 2021

Week starting	Population*			New cases^†^					New hospitalizations^§^							
	Average no. fully vaccinated^¶^	Average no. unvaccinated	Full vaccination coverage, %	Fully vaccinated		Unvaccinated		Estimated vaccine effectiveness, %	Fully vaccinated			Unvaccinated			Estimated vaccine effectiveness, %	
				No.	Rate*	No.	Rate*		No.	Rate*	No.		Rate*		
May 3	6,255,275	5,367,527	40.4	700	1.60	7,387	19.66	91.7	154	0.35	1,478		3.93	95.3	
May 10	6,948,727	4,938,120	44.9	589	1.21	5,839	16.89	92.7	149	0.31	1,145		3.31	95.0	
May 17	7,641,098	4,642,464	49.4	555	1.04	4,106	12.63	91.9	134	0.25	968		2.98	96.2	
May 24	8,222,099	4,444,612	53.1	431	0.75	2,757	8.86	92.0	140	0.24	748		2.40	93.8	
May 31	8,691,229	4,289,385	56.2	364	0.60	2,092	6.97	91.6	87	0.14	549		1.83	95.1	
Jun 7	9,034,873	4,226,865	58.4	341	0.54	1,504	5.08	89.7	95	0.15	448		1.51	93.3	
Jun 14	9,272,840	4,165,878	59.9	340	0.52	1,233	4.23	87.9	88	0.14	324		1.11	91.9	
Jun 21	9,516,612	4,022,274	61.5	396	0.59	1,201	4.27	85.8	60	0.09	283		1.01	94.6	
Jun 28	9,747,395	3,913,256	63.0	535	0.78	1,421	5.19	83.8	69	0.10	288		1.05	93.9	
Jul 5	9,911,987	3,870,504	64.1	928	1.34	2,223	8.20	82.4	72	0.10	270		1.00	94.4	
Jul 12	10,034,269	3,818,600	64.8	1,703	2.42	3,242	12.13	78.2	89	0.13	340		1.27	94.8	
Jul 19	10,135,322	3,742,197	65.5	2,793	3.94	5,500	21.00	79.8	134	0.19	467		1.78	95.3	
**Total**	**—**	**—**	**—**	**9,675**	**1.31**	**38,505**	**10.69**	**—**	**1,271**	**0.17**	**7,308**		**2.03**	**—**	

* Population sizes fully vaccinated and unvaccinated were computed daily. For display purposes, the average populations fully vaccinated and unvaccinated are shown for each week. Rate calculations were conducted using daily population sizes and are expressed per 100,000 person-days. Persons partially vaccinated were excluded from analyses.

^†^ New cases were defined as a new positive SARS-CoV-2 nucleic acid amplification test or antigen test result, not within 90 days of a previous positive result, reported to the Electronic Clinical Laboratory Reporting System, which collects all reportable COVID-19 test results in New York.

^§^ New hospitalizations were determined by a report of a hospital admission with a confirmed COVID-19 diagnosis, entered into the Health Electronic Response Data System, which includes a statewide, daily electronic survey of all inpatient facilities in New York.

^¶^ Persons were determined to be fully vaccinated following 14 days after final vaccine-series dose receipt, per the Citywide Immunization Registry and the New York State Immunization Information System, which collect and store all COVID-19 vaccine receipt data by providers for persons residing in New York City and the rest of New York, respectively.

**FIGURE 1:**
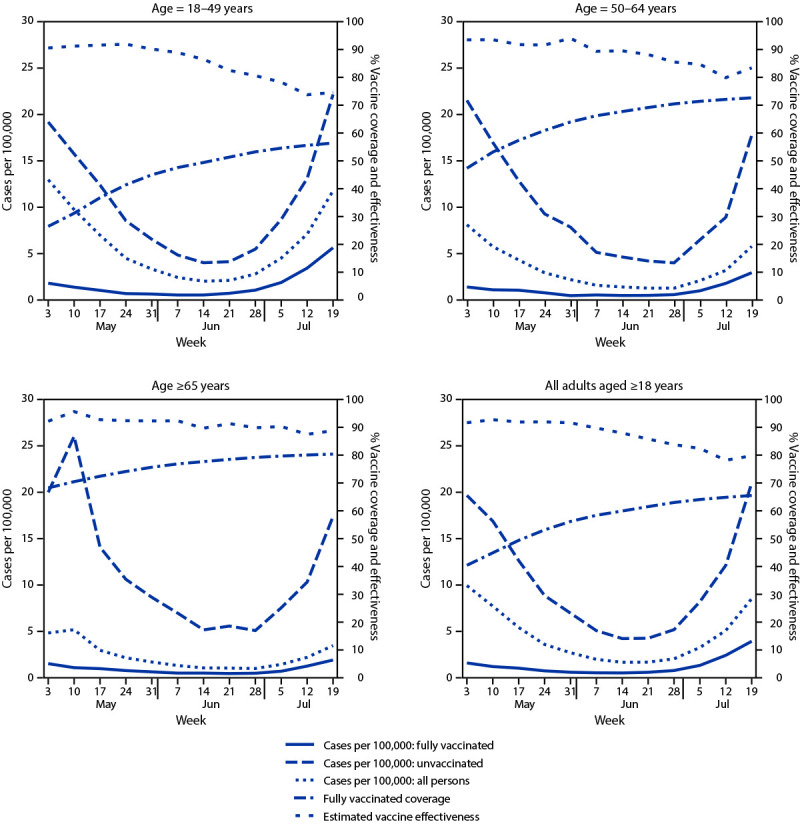
New COVID-19 cases among fully vaccinated and unvaccinated adults, vaccine coverage, and estimated vaccine effectiveness, by age — New York, May 3–July 25, 2021

A total of 1,271 new COVID-19 hospitalizations (0.17 per 100,000 person-days) occurred among fully vaccinated adults, compared with 7,308 (2.03 per 100,000 person-days) among unvaccinated adults (Table). Hospitalization rates generally declined through the week of July 5, but increased the weeks of July 12 and July 19, and were higher among fully vaccinated and unvaccinated persons aged ≥65 years compared with younger age groups (Figure 2). Age group–specific estimated VE against hospitalization remained stable, ranging from 90.8% to 97.5% for persons aged 18–49 years, from 92.4% to 97.0% for persons aged 50–64 years, and from 92.3% to 96.1% for persons aged ≥65 years. During May 3–July 25, the overall, age-adjusted VE against hospitalization was generally stable from 91.9% to 95.3% (Figure 2) (Table). The ratio of hospitalizations to cases was moderately lower among fully vaccinated (13.1 hospitalizations per 100 cases) compared with unvaccinated (19.0 hospitalizations per 100 cases) groups.

**FIGURE 2:**
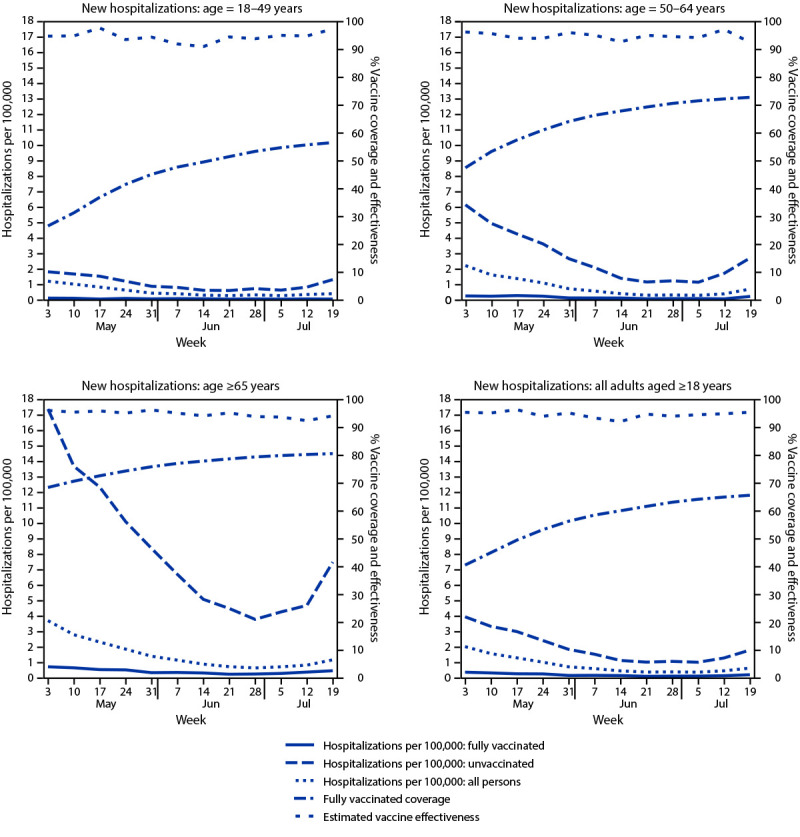
New hospitalizations with laboratory-confirmed COVID-19 among fully vaccinated and unvaccinated adults, vaccine coverage, and estimated vaccine effectiveness, by age — New York, May 3–July 25, 2021

## Discussion

In this study, current COVID-19 vaccines were highly effective against hospitalization (VE >90%) for fully vaccinated New York residents, even during a period during which prevalence of the Delta variant increased from <2% to >80% in the U.S. region that includes New York, societal public health restrictions eased,^§§^ and adult full-vaccine coverage in New York neared 65%. However, during the assessed period, rates of new cases increased among both unvaccinated and fully vaccinated adults, with lower relative rates among fully vaccinated persons. Moreover, VE against new infection declined from 91.7% to 79.8%. To reduce new COVID-19 cases and hospitalizations, these findings support the implementation of a layered approach centered on vaccination, as well as other prevention strategies.

The findings from this study are consistent with those observed in other countries. Israel has reported 90% VE for the Pfizer-BioNTech vaccine against hospitalization; however, a decline in VE against new diagnosed infections occurred during June 20–July 17 (decreasing to <65%) (*5*). Another study in the United Kingdom found higher VE against infection with the Delta variant for Pfizer-BioNTech (88%), which was lower than VE against the B.1.1.7 (Alpha) variant (94%) (*6*).

The factors driving the apparent changes in VE, including variations by age, are uncertain. Changes in immune protection from current vaccine product dosing regimens are under investigation,^¶¶^ with additional doses being considered (*7*). Increased Delta variant viral load might underpin its increased transmissibility and could potentially lead to reduced vaccine-induced protection from infection (*8*). Further, variations from clinical trial findings could be because the trials were conducted during a period before the emergence of new variants and when nonpharmaceutical intervention strategies (e.g., wearing masks and physically distancing) were more stringently implemented, potentially lessening the amount of virus to which persons were exposed. Other factors that could influence VE include indirect protective effects of unvaccinated persons by vaccinated persons and an increasing proportion of unvaccinated persons acquiring some level of immunity through infection (*9*).

The findings in this report are subject to at least six limitations. First, although limiting the analysis period to after universal adult vaccine eligibility and age stratification likely helped to reduce biases, residual differences between fully vaccinated and unvaccinated groups have the potential to reduce estimated VE. Second, the analysis excluded partially vaccinated persons, to robustly assess VE for fully vaccinated compared with that of unvaccinated persons. A supplementary sensitivity analysis that included partially vaccinated persons as unvaccinated yielded conservative VE for laboratory-confirmed infection (declining from 88.7% to 72.1%) and for hospitalizations (ranging from 89.7% to 93.0%). Third, exact algorithms were used to link databases; some persons were possibly not linked because matching variables were entered differently in the respective systems. Fourth, this study did not estimate VE by vaccine product, and persons were categorized fully vaccinated at 14 days after final dose, per CDC definitions; however, the Janssen vaccine might have higher efficacy at 28 days.*** Given that Janssen vaccine recipients accounted for 9% of fully vaccinated persons and the observed time period from full vaccination to infection (median 85 days), this would minimally affect the findings. Fifth, information on reasons for testing and hospitalization, including symptoms, was limited. However, a supplementary analysis found that among 1,271 fully vaccinated adults and 7,308 unvaccinated adults, 545 (42.9%) and 4,245 (58.1%), respectively, were reported to have been admitted for COVID-19 by hospital staff members using nonstandardized definitions. A sensitivity analysis of hospitalization VE limited to those admitted for COVID-19, found similar results (VE range = 93.9%–97.4%), suggesting that the extent of bias was limited. Finally, data were too sparse to reliably estimate VE for COVID-19-related deaths.

This study’s findings suggest currently available vaccines have high effectiveness for preventing laboratory-confirmed SARS-CoV-2 infection and COVID-19 hospitalization. However, VE against infection appears to have declined in recent months in New York, coinciding with a period of easing societal public health restrictions^†††^ and increasing Delta variant circulation (*8*). These findings support a multipronged approach to reducing new COVID-19 hospitalizations and cases, centered on vaccination, and including other approaches such as masking and physical distancing.

**Summary**

**What is already known about this topic?**

Real-world studies of population-level vaccine effectiveness against laboratory-confirmed SARS-CoV-2 infection and COVID-19 hospitalizations are limited in the United States.

**What is added by this report?**

During May 3–July 25, 2021, the overall age-adjusted vaccine effectiveness against hospitalization in New York was relatively stable (91.9%–95.3%). The overall age-adjusted vaccine effectiveness against infection for all New York adults declined from 91.7% to 79.8%.

**What are the implications for public health practice?**

These findings support the implementation of multicomponent approach to controlling the pandemic, centered on vaccination, as well as other prevention strategies such as masking and physical distancing.

## Footnotes

* As of the publication date of this report, COVID-19 vaccines by Pfizer-BioNTech, Moderna, and Janssen (Johnson & Johnson) have been authorized by the Food and Drug Administration under Emergency Use Authorization.

† Final dose was the second dose for Pfizer-BioNTech and Moderna vaccines, first dose for Janssen vaccine.

§
https://www.governor.ny.gov/news/governor-cuomo-announces-new-yorkers-30-years-age-and-older-will-be-eligible-receive-covid-19

¶ The total adult state population that was unvaccinated was calculated as the total U.S. Census population, minus fully or partially vaccinated persons. Persons who were partially vaccinated were defined as those who initiated a vaccine series but did not complete it or were within 14 days after completion.

** For both outcomes, VE at each week and age group was calculated as 1-(Rate_vaccinated_ / Rate_unvaccinated_).

†† The percentage tested ≥7 days from being fully vaccinated was included to inform possible undiagnosed infection before full vaccination was achieved.

§§
https://covid.cdc.gov/covid-data-tracker/#variant-proportions

¶¶
https://www.medrxiv.org/content/10.1101/2021.07.28.21261159v1

***
https://www.fda.gov/media/146338/download

†††
https://coronavirus.jhu.edu/data/state-timeline/new-confirmed-cases/new-york/205
